# The Synergy Reinforcement Effect of Sm_0.85_Zn_0.15_MnO_3_ and ZrMgMo_3_O_12_ on Sm_0.85_Zn_0.15_MnO_3_-ZrMgMo_3_O_12_/Al-20Si Composites

**DOI:** 10.3390/ma17112494

**Published:** 2024-05-22

**Authors:** Kaidong Li, Bo Ren, Zhongxia Liu, Guopeng Zhang, Bin Cai, Yunjia Shi, Hai Huang

**Affiliations:** 1 Key Laboratory of Material Physics of Ministry of Education, School of Physics, Zhengzhou University, Zhengzhou 450052, China; lkd2080819319@gs.zzu.edu.cn (K.L.);; 2 Faculty of Engineering, Huanghe Science and Technology College, No. 666 Zijingshan South Road, Zhengzhou 450061, China

**Keywords:** Sm_0.85_Zn_0.15_MnO_3_ NTE ceramic, ZrMgMo_3_O_12_ NTE ceramic, aluminum matrix composites, compressive mechanical properties, coefficient of thermal expansion

## Abstract

Negative thermal expansion (NTE) ceramics Sm_0.85_Zn_0.15_MnO_3_ (SZMO) and ZrMgMo_3_O_12_ (ZMMO) were selected to prepare Sm_0.85_Zn_0.15_MnO_3_-ZrMgMo_3_O_12_/Al-20Si (SZMO-ZMMO/Al-20Si) composites using ball milling and vacuum heating-press sintering processes in this study. The synergistic effect of the SZMO and ZMMO NTE ceramic reinforcements on the microstructure, mechanical properties, and coefficient of thermal expansion (CTE) of the composites was investigated. The results show that the processes of ball milling and sintering did not induce the decomposition of SZMO or ZMMO NTE ceramic reinforcements, nor did they promote a reaction between the Al-20Si matrix and SZMO or ZMMO NTE ceramic reinforcements. However, the excessive addition of SZMO and ZMMO NTE ceramics led to their aggregation within the composite. Adding a small amount of SZMO in combination with ZMMO effectively increased hardness and yield strength while reducing CTE in the Al-20Si alloy. The improvement in strength was primarily provided by SZMO, while the inhibition effect on CTE was primarily provided by ZMMO. An evaluation parameter denoted as α was proposed to evaluate the synergy effects of SZMO and ZMMO NTE ceramic reinforcements on the mechanical properties and CTE of the composites. Based on this parameter, among all composites fabricated, adding 2.5 vol% SZMO NTE ceramic and 10 vol% ZMMO NTE ceramic resulted in an optimal balance between CTE and strength for these composites with a compressive yield strength of 349.72 MPa and a CTE of 12.55 × 10^−6^/K, representing a significant increase in yield strength by 79.20% compared to that of Al-20Si alloy along with a notable reduction in CTE by 26.44%.

## 1. Introduction

Thermal expansion is a fundamental physical law that governs most materials in nature, causing them to expand or contract with temperature. This can lead to dimensional changes and thermal stress cracking in certain precision instruments and equipment (such as inertial navigation devices), reducing their measurement accuracy and service life. To mitigate these issues, it is critical to select materials with lower CTE for inertial navigation devices.

After continuous development in recent years, materials used in inertial navigation devices have undergone three iterations, from aluminum alloy to beryllium alloy and now to aluminum matrix composites. Aluminum matrix composites have attracted the attention of many researchers due to their advantages of low cost, low density, high specific strength, high ductility, and excellent processability. The reinforcements added to the traditional aluminum matrix composites are typically categorized into particles (such as SiC [1,2,3,4,5,6,7], Al_2_O_3_ [8,9], B_4_C [10,11], TiC [12,13], graphite [14,15], diamond [16,17], graphene [18,19], and others), whiskers [20,21], fibers [22,23], and nanomaterials [24]. Among these options, particle reinforcement plays a predominant role in improving the dimensional stability of composites. It has been demonstrated that SiC/Al composites possess adjustable CTE within the range of 6.9 × 10^−6^/K to 9.7 × 10^−6^/K by varying the amount of added SiC particles and the kind of aluminum matrix to meet various engineering requirements [25]. Consequently, they exhibit excellent dimensional stability and are widely used in inertial instruments. However, in order to maintain the excellent thermal expansion performance of SiC/Al composites, a substantial amount of reinforcement needs to be incorporated [26], which seriously deteriorates the processability and toughness of SiC/Al composites [27,28]. Therefore, it is critical to explore novel reinforcements for developing aluminum matrix composites with enhanced dimensional stability and improved mechanical properties.

NTE materials [29,30,31,32,33,34] are characterized by contracting instead of expanding when heated, resulting in a negative CTE. The thermal contraction of NTE materials can be used to compensate for the thermal expansion of the metallic matrix caused by temperature fluctuations. By incorporating the NTE ceramics into a metallic matrix with positive CTE, the undesired expansions and contractions caused by temperature fluctuations can be effectively counteracted, thereby enhancing the stability and durability of composites under varying environmental conditions. Consequently, NTE ceramics present a promising option for reinforcing high-dimensional, stable metallic matrix composites. Some NTE ceramics had been used to fabricate metallic matrix composites [35,36,37,38,39] and demonstrated that incorporating a small amount of NTE ceramics can cause a significant decrease in the CTE of composites, which is highly beneficial for the improvement of dimensional stability. However, some unfavorable characteristics, such as structural instability [36], limited NTE temperature range [37], hygroscopicity [38], easy reaction with metallic matrix alloy [39], etc., may alter the CTE of the NTE ceramic-reinforced metallic matrix composites in the actual service process, thereby affecting both the mechanical properties and thermal expansion properties of the prepared composites. Therefore, selecting structurally stable NTE ceramic reinforcements is a key consideration in developing dimensionally stable metallic matrix composites.

The oxide NTE ceramic ZMMO [40] has a stable orthorhombic structure and does not undergo phase change or water absorption. It exhibits a negative CTE (−3.73 × 10^−6^/K) over a wide temperature range (294–775 K). The investigations conducted by Yang et al. [41,42] have demonstrated its potential as reinforcement in composites for inertial navigation devices. However, the significant difference in CTE between ZMMO reinforcement and the 2024Al matrix results in high thermal mismatch stress at the ZMMO/α-Al interface, which may lead to composites cracking. Consequently, increasing the amount of ZMMO added to reduce the CTE of ZMMO/2024Al composites becomes challenging due to this issue. Moreover, the flexible orthorhombic frame structure of ZMMO leads to lower hardness (205 HV) and elastic modulus (49.45 GPa) [43]. As a result, it has limited strengthening effects on the composite and is not conducive to maintaining dimensional stability under stress conditions. Therefore, it is imperative to strengthen the NTE reinforcement in order to synergistically improve both the mechanical properties and thermal expansion properties of ZMMO reinforced aluminum matrix composites.

The SZMO [44] ceramic is another highly structurally stable NTE ceramic, with an adjustable CTE from −5.10 × 10^−6^/K to 5.58 × 10^−6^/K, depending on its porosity in the microstructure. Its perovskite structure also provides high hardness (387 HV), making it a potential reinforcement for fabricating metallic composites used in inertial navigation devices. However, the NTE effect of SZMO is only exhibited when the porosity in the microstructure exceeds 9.5%, as it results from the coupling effect between high porosity and microstructural NTE caused by anisotropic thermal expansion of crystal grains and intercrystalline pores. As the reduction in particle size may decrease its porosity, this special NTE mechanism may weaken the thermal expansion compensation effect of fine SZMO reinforcement on the metallic matrix. Nevertheless, due to their exceptional strength and hardness characteristics, SZMO NTE ceramic particles can effectively strengthen aluminum alloys through dispersion strengthening mechanisms. Therefore, SZMO was experimentally selected in this study to be combined with ZMMO and added into an Al-20Si matrix for fabricating ZMMO-SZMO reinforced aluminum matrix composites, aiming to enhance the strengthening effect of NTE reinforcements on aluminum matrix while maintaining low CTE values in the resulting composites. Consequently, ZMMO-SZMO/Al-20Si composites with varying contents of ZMMO and SZMO were prepared by ball milling and vacuum heating-press sintering techniques. The effects of reinforcement content on the microstructure, mechanical properties, and thermal expansion properties of the composites were investigated. Furthermore, the synergistic effect of ZMMO and SZMO NTE ceramic reinforcements on mechanical properties and CTE was analyzed.

## 2. Experimental Materials and Procedures

### 2.1. Experimental Materials

Commercial Al-20Si powder with a nominal size of 10–20 µm and chemical composition (mass fraction, %) of 20% Si, 0.2% Fe, and Al balance was supplied by Qing He Yao Xie Metal Materials Co. Ltd. (Xingtai, Hebei, China). The ZMMO powder with a nominal size of 0.5–1 μm and the SZMO powder with a nominal size of 1–3 μm were synthesized in the author’s laboratory. The synthesis details of the ZMMO and SZMO reinforcements have been previously reported [43,44].

### 2.2. Preparation of Composites

Six SZMO-ZMMO/Al-20Si composites (Samples C_1_ to C_6_), incorporating a combined NTE ceramic reinforcement content ranging from 6.5 to 13.5 vol%, were fabricated via a ball milling and vacuum heating-press sintering process. The additional amount of two kinds of NTE ceramic reinforcements is shown in Table 1. It should be noted that SZMO has a relatively high density of 8.6 g/cm^3^, and excessive incorporation into the composite would lead to increased composite density. Therefore, considering the requirement for lightweight composites, only 1.5–3.5 vol% SZMO was added in this study. Initially, the SZMO NTE ceramic powders, ZMMO NTE ceramic powders, and commercial Al-20Si alloy powders were weighed according to the stoichiometric ratio of the composite and placed in a zirconia ball milling tank along with zirconia balls at a ratio of 10:1 to obtain the powder mixture of commercial Al-20Si alloy, SZMO, and ZMMO NTE ceramics. Subsequently, the powder mixture was ball milled for 8 h using a QM-WX 4 planetary ball mill at a speed of 300 rpm in an anhydrous ethanol medium. Afterwards, the milled powder mixture of SZMO-ZMMO/Al-20Si powder was dried for 18 h at 55 °C in a DZF-6050 vacuum drying oven. Finally, the dried powder mixture of SZMO-ZMMO/Al-20Si was heated to 555 °C at a heating rate of 5 °C/min under a vacuum atmosphere at 60 MPa pressure for sintering purposes using the vacuum heating-press sintering method. The sintering process lasted for 3 h and the sintered composite bulks were subsequently cooled to room temperature within the furnace. The sintering temperature of 555 °C was determined based on the previous research conducted by our group and has been identified as the optimal temperature for achieving maximum densification of the composite. For comparison purposes, three reference samples were also prepared by ball milling and a heating-press sintering process similar to that employed for SZMO-ZMMO/Al-20Si composites. These reference samples include bulk Al-20Si alloy (Sample A), ZMMO/Al-20Si composites (Sample B_1_), and SZMO/Al-20Si composites (Sample B_2_).

### 2.3. Microstructure Characterization of Composites

The phases of the mixed powders and bulk composites, including reference samples and experimental samples, were determined using an X-ray diffractometer (XRD) (PANalytical, Almelo, Holland) equipped with Cu Kα rays (λ = 0.15406 nm). The 2*θ* scan range was set from 10 to 80° with a step size of 0.01°. The microstructure of reference samples and experimental samples was observed by a JSM-6700 F cold field emission scanning electron microscope (SEM) manufactured by JEOL (Tokyo, Japan). The phase composition was analyzed by an energy dispersive spectrometer (EDS) attached to the SEM (Tokyo, Japan).

### 2.4. Measurement of Mechanical Properties

The hardness of reference samples and experimental samples was measured by an HVS-50 digital display (Taiming Optical instrument, Shanghai, China). Vickers hardness tester using a test load of 9.8 N and a holding time of 15 s. A total of 10 points were measured for each sample, and the average value was taken as the hardness of the sample. The compression mechanical properties were evaluated by the MTS 810 material testing machine (MTS, Minneapolis, MN, USA) using compressed samples with dimensions of 4 mm × 4 mm × 8 mm at a loading rate of 0.2 mm/min. Three replicates were performed, and the compressive yield strength (*σ*_y_), compressive fracture strength (*σ*_f_), and compressive fracture strain (*ε*_f_) were evaluated based on the average of three test results.

### 2.5. Measurement of CTE

The CTEs of reference samples and experimental samples with dimensions of 4 mm × 4 mm × 8 mm were measured using a LINSEIS DIL L76 thermal dilatometer (LINSEIS, Selb, Bavaria, Germany) at a heating rate of 5 °C/min over a temperature range from room temperature to 400 °C. The CTE of measured composites was estimated according to Equation (1) [42], which defined it as the slope of the relative length curve of the measured composites with increasing temperature:

(1) $$CTE=(\Delta L-K)/[L_{1}*\left(T_{2}-T_{1}\right)]\;$$

where *L*_2_ and *L*_1_ present the length of the measured composites at temperature *T*_2_ and *T*_1,_ respectively, Δ*L* presents the change in relative length from temperature *T*_1_ to *T*_2_ for the measured composites, and *K* is the system compensation value at temperature *T*_2_, which can be estimated by Equation (2) [42]:

(2) $$K={\Delta L}_{ref}-{CTE}_{ref}*L_{ref}\left(T_{2}-T_{1}\right)$$

where *L*_ref_ is the length of the quartz standard sample at temperature *T*_1_, Δ*L*_ref_ is the change value in the relative length of the quartz standard sample after raising its temperature from *T*_1_ to *T*_2_ and *CTE*_ref_ is the average linear CTE of the quartz standard sample between temperatures *T*_1_ and *T*_2_. Three replicates were performed for each measurement, and the average CTEs were reported.

## 3. Results and Discussion

### 3.1. Microstructural Characterization of Composites

**Figure 1:**
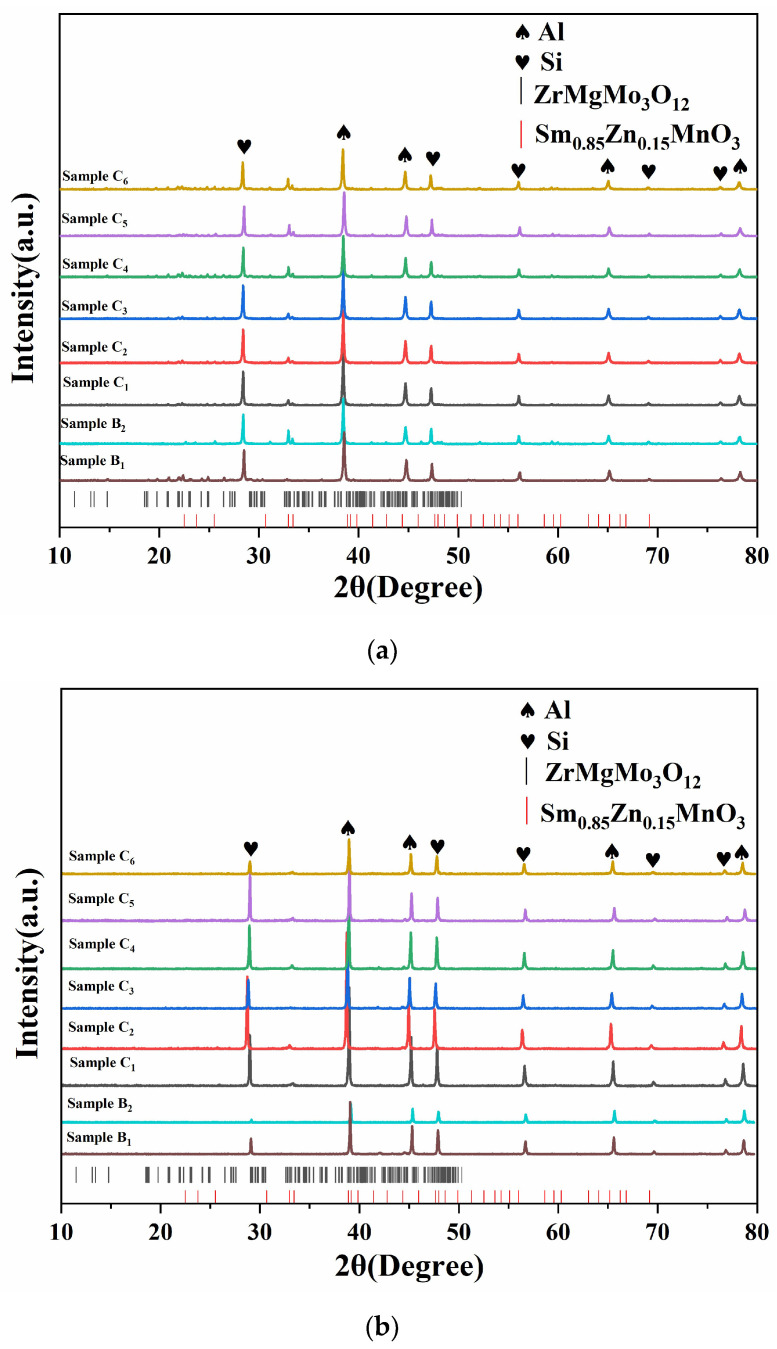
XRD patterns of mixed powders (**a**) and bulk composites of SZMO-ZMMO/Al-20Si with varying ZMMO and SZMO reinforcement contents (**b**).

Figure 1 represents the typical XRD pattern of mixed powders and bulk SZMO-ZMMO/Al-20Si with various NTE ceramic reinforcement contents. It was evident that both the mixed powders and bulk composites exclusively consist of α-Al, primary Si, ZMMO, and SZMO NTE ceramic reinforcements. The aforementioned observation indicated that ball milling and sintering did not induce the decomposition of SZMO or ZMMO NTE ceramic reinforcements, nor did they promote a reaction between the Al-20Si matrix and SZMO or ZMMO NTE ceramic reinforcements, thereby demonstrating excellent phase stability.

**Figure 2:**
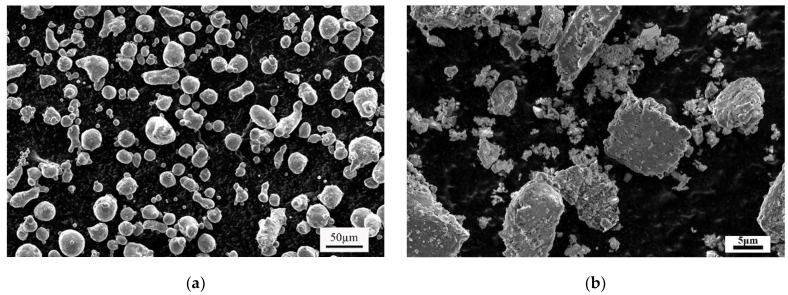
Morphologies of as-received Al-20Si powders (**a**) SZMO-ZMMO/Al-20Si mixed powders (**b**) after ball milling.

Figure 2 represents the typical morphologies of mixed powders. As shown in Figure 2, the morphology of Al-20Si powders underwent a transformation from nearly spherical particles (Figure 2a) to lamellar and flat particles (Figure 2b). Additionally, a significant number of fine SZMO and ZMMO NTE ceramic reinforcement particles were embedded on the surface of Al-20Si alloy particles (Figure 2b), due to the serious plastic deformation and work hardening of Al-20Si particles experienced during the ball milling process. This observation suggested that the toughed Al-20Si particles effectively capture brittle ZMMO and SZMO NTE ceramic particles in the process of ball milling, resulting in their dispersion within the Al-20Si alloy matrix.

**Figure 3:**
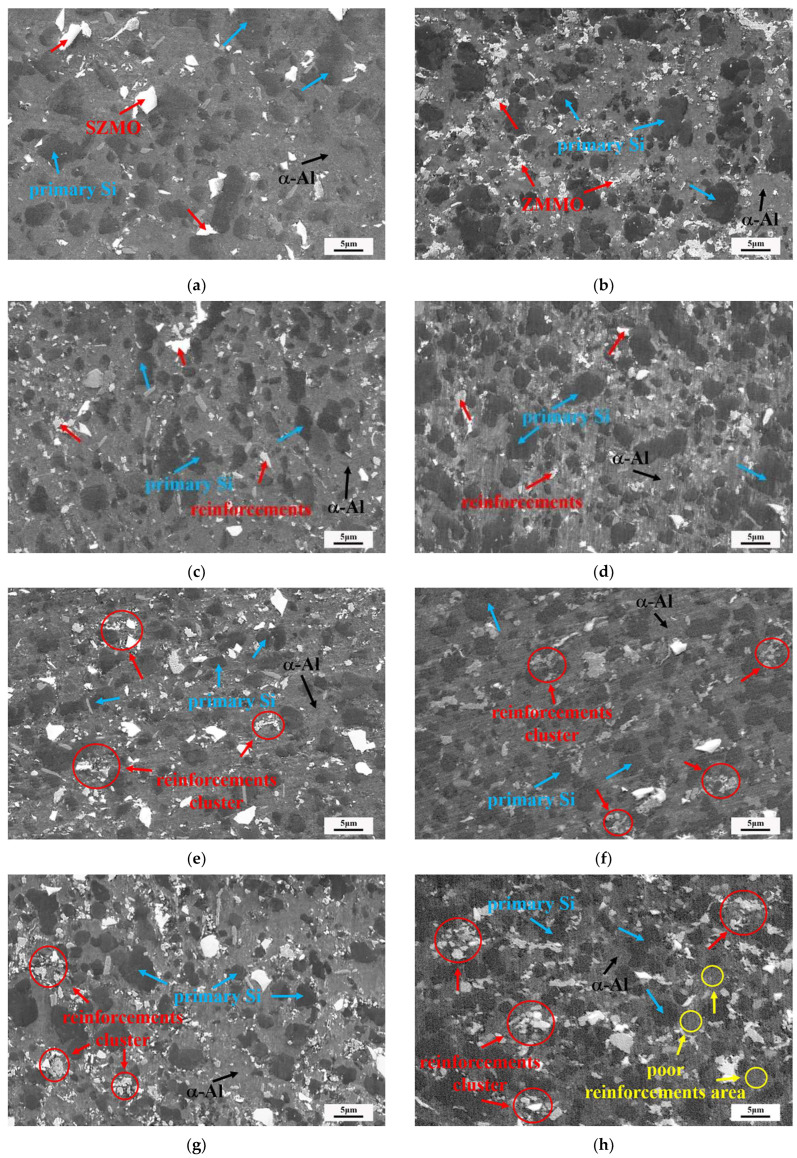
Microstructures of SZMO-ZMMO/Al-20Si composites: (**a**) B_1_; (**b**) B_2_; (**c**) C_1_; (**d**) C_2_; (**e**) C_3_; (**f**) C_4_; (**g**) C_5_; (**h**) C_6._

**Figure 4:**
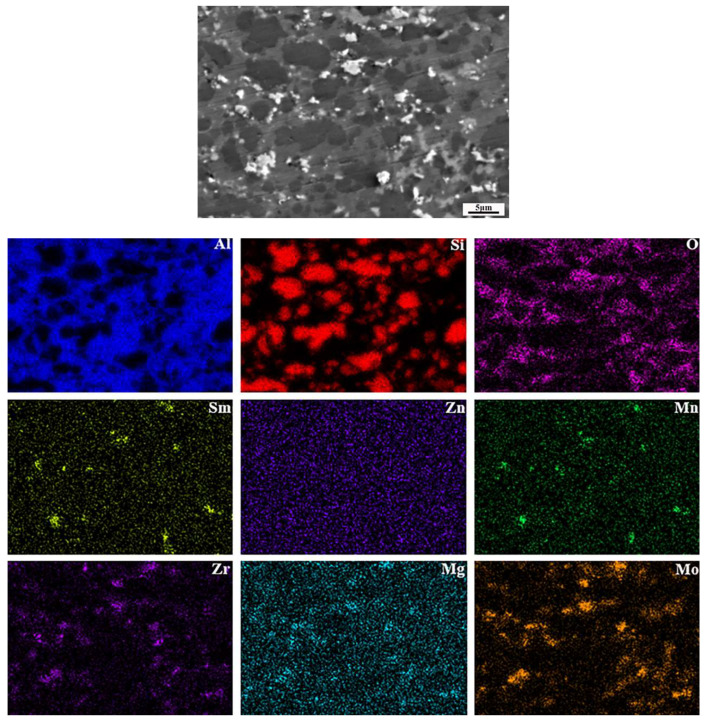
The SEM-EDS elemental mapping of sample C_4_.

The microstructure of bulk SZMO-ZMMO/Al-20Si composites with varying ZMMO and SZMO reinforcement contents was observed using SEM, and the observation results are represented in Figure 3. It can be seen that all composites are composed of a gray matrix along with bright white and black second phases. In order to identify the phases in the microstructure more clearly, a representative sample of C_4_ was selected for analysis with SEM-EDS elemental mapping, and the results are represented in Figure 4. By combining XRD and EDS analyses in Figure 1 and Figure 4, it can be determined that the composites were composed of black primary Si, dark gray α-Al, and bright white SZMO and ZMMO NTE ceramic reinforcement phases. It is worth noting that although SZMO and ZMMO reinforcements could be distinguished from the SEM-EDS elemental mapping as shown in Figure 4, they were difficult to differentiate within the microstructure in Figure 3 due to their similar color appearance. Therefore, they are grouped in the subsequent analysis of their distribution.

Figure 3 and Figure 4 clearly demonstrate the distribution of SZMO and ZMMO NTE ceramic reinforcements. Referring to Figure 3, the uniformity of NTE ceramic reinforcement distribution rose and then declined with the increase in SZMO reinforcement for a certain ZMMO content. The 2.5 vol% SZMO NTE ceramic (C_2_ and C_5_) resulted in the most uniform distribution of reinforcements (Figure 3d,g). Additionally, the total reinforcement content of SZMO and ZMMO was another factor affecting the uniformity of reinforcement distribution. When the total reinforcement content was below 7.5 vol% (Figure 3c,d), the composites exhibited a dispersed distribution of SZMO and ZMMO reinforcements in the α-Al matrix. However, when the total reinforcement content exceeded 8.5 vol% (Figure 3e–g), a slight agglomeration phenomenon was observed. Furthermore, when the reinforcement content reached 13.5 vol% (Figure 3h), a significant agglomeration phenomenon occurred, characterized by large clusters of SZMO and ZMMO reinforcements in the α-Al matrix along with distinct regions of rich—(SZMO, ZMMO) and poor—(SZMO, ZMMO). These results indicated that the content of NTE ceramic reinforcements affects its distribution in the α-Al matrix of composites. Higher reinforcement content enhanced the agglomeration in the α-Al matrix due to an increased collision probability among zirconia grinding balls, SZMO and ZMMO NTE ceramic particles during the mechanical milling process, resulting in finer reinforcement particles compared to those present at lower content reinforcements. Fine SZMO and ZMMO NTE ceramic particles possessed high surface energy, which promoted their agglomeration, which led to serious SZMO and ZMMO particle segregation within the composite.

### 3.2. Hardness of SZMO-ZMMO/Al-20Si Composites

**Figure 5:**
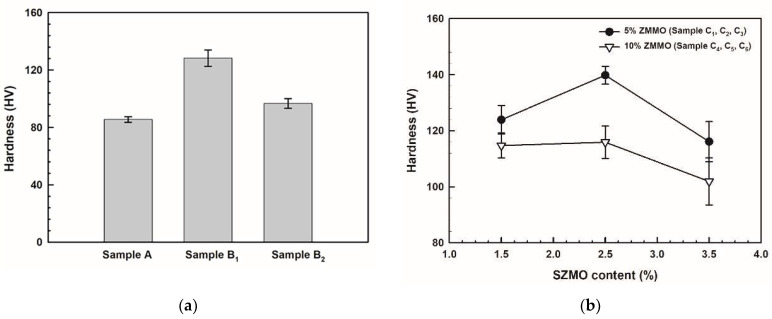
The hardness of (**a**) Reference samples (Sample A, Samples B_1_, and B_2_) and (**b**) Experimental samples (C_1_ to C_6_, SZMO-ZMMO/Al-20Si composites with varying reinforcement contents).

In order to evaluate the effect of reinforcement content on the mechanical properties of SZMO-ZMMO/Al-20Si composites, Vickers hardness measurements were conducted for SZMO-ZMMO/Al-20Si composites with varying reinforcement content, and the results are represented in Figure 5. For comparison purposes, the hardness values of Al-20Si alloy (Sample A), SZMO/Al-20Si composite (Sample B_1_) and ZMMO/Al-20Si composite (Sample B_2_) were also included in Figure 5a. Referring to Figure 5a, it can be observed that singly adding 10 vol% ZMMO increased the hardness of the Al-20Si alloy by a mere 12.8%, while singly incorporating 5 vol% SZMO led to a significant hardness enhancement of 50.1%. This suggested that SZMO NTE ceramic exhibited a robust strengthening effect, whereas ZMMO NTE ceramic had a relatively weaker influence on the strength of Al-20Si alloys. Referring to Figure 5b, it is evident that the hardness of SZMO-ZMMO/Al-20Si composites surpassed that of the bare Al-20Si matrix (Sample A), indicating a clear reinforcing effect resulting from the combined addition of SZMO-ZMMO NTE ceramic reinforcements. With an increase in SZMO NTE ceramic content for a certain ZMMO NTE ceramic content, the hardness initially rose and then declined. However, increasing ZMMO NTE ceramic content from 5 vol% to 10 vol% led to a decrease in hardness. This demonstrated that adding a small amount of SZMO NTE ceramics significantly improved the reinforcing effect provided by ZMMO NTE ceramics on the Al-20Si matrix alloy. Therefore, the combined incorporation of both SZMO and ZMMO NTE ceramics yielded greater synergy-reinforcing effects compared to solely adding ZMMO NTE ceramics alone. These findings suggested that SZMO NTE ceramics effectively compensated for the relatively low reinforcement effect observed with ZMMO NTE ceramics in ZMMO/Al-20Si composites. However, the results of the SZMO-SZMO/Al-20Si composite with 10 vol% ZMMO had lower hardness than those with 5 vol% ZMMO, inferring that the high content of ZMMO reduced the compensation effect of SZMO on ZMMO strengthening. The highest hardness was achieved when combining 2.5 vol% SZMO and 5 vol% ZMMO (sample C_2_), resulting in a significant increase of 63.5% compared to the bare Al-20Si alloy (sample A) and a notable increase of 43.7% compared to the ZMMO/Al-20Si composite (sample B_2_).

### 3.3. Compressive Properties of SZMO-ZMMO/Al-20Si Composites

**Figure 6:**
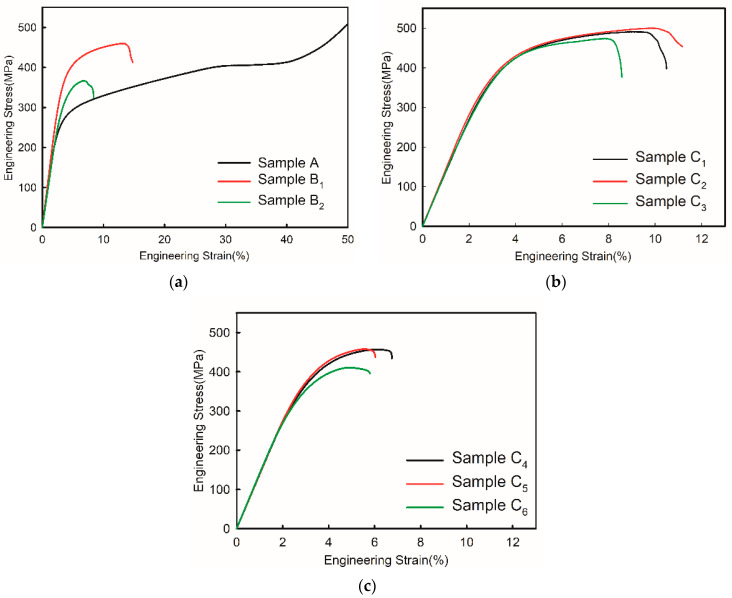
Compressive stress–strain curves of (**a**) Reference samples (Sample A, Samples B_1_ and B_2_), (**b**) Experimental samples with 5 vol% ZMMO (Samples C_1_, C_2_, C_3_) and (**c**) Experimental samples with 10 vol% ZMMO (Samples C_4_, C_5_, C_6_).

**Figure 7:**
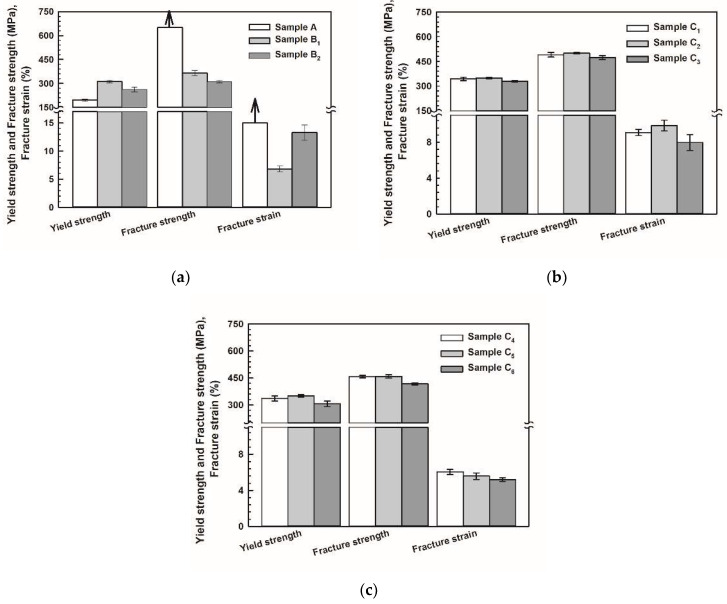
Compressive yield strength, compressive fracture strength and compressive fracture strain of (**a**) Reference samples (Sample A, Samples B_1_ and B_2_) (The arrow in the figure indicates that the sample didn’t fail and the estimated data was beyond the range of the *Y*-axis). (**b**) Experimental samples with 5 vol% ZMMO (Samples C_1_, C_2_, C_3_) and (**c**) Experimental samples with 10 vol% ZMMO (Samples C_4_, C_5_, C_6_).

In order to further study the synergy reinforcement effect of SZMO-ZMMO reinforcements on the mechanical properties of composites, the compressive mechanical properties of SZMO-ZMMO/Al-20Si composites with various reinforcement contents were measured by a room-temperature compression test. Figure 6 shows the compressive stress–strain curves of SZMO-ZMMO/Al-20Si composites. For comparison purposes, the compressive stress–strain curves of reference samples are also represented in Figure 6. Referring to Figure 6, the incorporation of SZMO and ZMMO NTE ceramics increased the strength but decreased the ductility. It showed that the addition of high-content SZMO and ZMMO NTE ceramic reinforcements decreased the plastic deformation ability of SZMO-ZMMO/Al-20Si composites. To evaluate the effect of SZMO and ZMMO NTE ceramics on the compressive mechanical properties, the compressive yield strength, compressive fracture strength, and compressive fracture strain of the SZMO-ZMMO/Al-20Si composites were estimated from Figure 6, and the results are shown in Figure 7. It can be seen that, similar to the evolution trend of hardness, the single addition of SZMO or ZMMO NTE ceramic could obviously strengthen the Al-20Si alloy. The yield strength increased from 195.2 MPa of Al-20Si (Sample A) to 311.6 MPa of SZMO/Al-20Si (Sample B_1_) and 263.1 MPa of ZMMO/Al-20Si (Sample B_2_), respectively, by 59.6% and 34.8%. The combined addition of SZMO and ZMMO NTE ceramics improved the strengthening effect of ZMMO NTE ceramics. The compressive yield strength and compressive fracture strength of the SZMO-ZMMO/Al-20Si composites first increased and then decreased with the increase in SZMO NTE ceramics. The SZMO-ZMMO/Al-20Si composites containing 2.5 vol% SZMO and 5 vol% ZMMO NTE ceramics (sample C_2_) had the highest compressive yield strength and compressive fracture strength, which were 350.76 MPa and 499.86 MPa, respectively. The compressive yield strength increased by 79.7% compared to the Al-20Si matrix alloy (Sample A) and by 33% compared to the ZMMO/Al-20Si composite (Sample B_2_). The compressive fracture strength increased by 60.5% compared to the ZMMO/Al-20Si composite (Sample B_2_). These results demonstrated once again that SZMO NTE ceramic exhibited a more significant reinforcing effect on Al-20Si alloys than ZMMO NTE ceramic. Adding a small amount of SZMO could compensate for the lower reinforcing effect of ZMMO on Al-20Si. However, the high content of ZMMO undermined the compensation effect of SZMO on the reinforcing effect of ZMMO on the Al-20Si alloy. Notably, only the yield strength of the Al-20Si bare alloy was compared in this study because the Al-20Si alloy exhibited exceptionally compressive ductility during the compression test, resulting in only deformation without fracture. Consequently, its compressive fracture strength was difficult to measure.

The improvement of mechanical properties of SZMO-ZMMO/Al-20Si composites mainly depends on the dispersion strengthening of SZMO and ZMMO NTE ceramic particles in the Al-20Si matrix alloy, the load transfer strengthening between α-Al and reinforcements (SZMO and ZMMO NTE ceramics), the thermal mismatch strengthening between α-Al and reinforcements (SZMO and ZMMO NTE ceramics), as well as the fine grain strengthening caused by the ball milling process. The dispersion-strengthening effect of SZMO and ZMMO NTE ceramic particles in Al-20Si is influenced by their respective mechanical properties. The low hardness and elastic modulus of ZMMO NTE ceramics due to their flexible orthorhombic frame structure make them prone to cracking under stress concentration caused by dislocation accumulation. Consequently, it exhibited less resistance to dislocation motion and resulted in a weak dispersion-strengthening effect. Due to the limited strengthening effect of ZMMO, when ZMMO was combined with SZMO, the deformation and cracking preferentially occurred at the ZMMO position rather than the SZMO position. Accordingly, the strength of the composite was predominantly determined by the strengthening effect of ZMMO rather than SZMO. As shown in Figure 7a, not only did the ZMMO/Al-20Si composite (Sample B_2_) exhibit low strength and ductility, but also the strength and ductility of SZMO-ZMMO/Al-20Si composites decreased with increasing content of ZMMO NTE ceramic particles, referring to Figure 7b,c. In contrast to ZMMO NTE ceramics, the high hardness and elastic modulus of SZMO NTE ceramics with a perovskite structure enabled them to be subjected to greater stress without cracking. Therefore, it could tolerate greater stress concentration at the SZMO/α-Al matrix interface without cracking, which led to stronger resistance to dislocation motion and a dispersion-strengthening effect. As shown in Figure 7, not only did singly adding SZMO NTE ceramics (Sample B_1_) result in higher yield strength and fracture strength compared to adding solely ZMMO NTE ceramics into Al-20Si alloy (Sample B_2_), referring to Figure 7a, but also the combined addition of both NTE ceramics led to high yield strength and fracture strength for the resulting SZMO-ZMMA/Al-20Si composites, referring to Figure 7b,c. The complementary reinforcing effect provided by adding a small amount of SZMO NTE ceramics was highly significant.

However, the complementary reinforcing effect of SZMO NTE ceramic reinforcements on ZMMO NTE ceramic reinforcements is closely related to their volume fraction, particle size, and distribution in the α-Al matrix. The high volume fraction, superior strength, and uniform distribution of reinforcements in the α-Al contributed to enhancing the dispersion-strengthening effect of NTE ceramic reinforcements. As aforementioned earlier, increasing the volume fraction of NTE ceramic reinforcements led to a higher collision probability among hard ZMMO, SZMO particles, and grinding balls, resulting in further refinement of ZMMO and SZMO NTE ceramic particles. Fine ZMMO and SZMO particles effectively impeded dislocation motion. Therefore, the enhanced complementary reinforcing effect of SZMO on ZMMO NTE ceramic particles arose from both dispersion strengthening caused by high-volume fraction NTE ceramic particles and fine grain strengthening resulting from fine SZMO and ZMMO NTE ceramic particles.

It is noted that an excessive amount of SZMO NTE ceramics led to a decrease in the strength of the SZMO-ZMMO/Al-20Si composite due to the agglomeration of SZMO-ZMMO reinforcements. As shown in Figure 3, SZMO and ZMMO NTE ceramic particles were evenly distributed in the α-Al matrix at a low reinforcement content (less than 7.5 vol%). However, when the content of reinforcement exceeded 8.5 vol%, the agglomeration of NTE ceramic reinforcements began to appear. Furthermore, when the reinforcement content reached 13.5 vol%, a significant agglomeration phenomenon was observed, which decreased their Orowan strengthening effect [45,46] and consequently reduced the strength of SZMO-ZMMO/Al-20Si composites. The variation in hardness with reinforcement content exhibited a similar trend to that observed in the hot pressing sintered Ni_0.05_Mo_3_Sb_5.4_Te_1.6_/Al_2_O_3_ composite, attributing to the aggregation of Al_2_O_3_ caused by a high content of Al_2_O_3_ [47]. Additionally, a significant difference in CTE between the Al-20Si matrix and ZMMO and SZMO NTE ceramic reinforcements resulted in large thermal residual stress at the reinforcements/α-Al interface. This caused deformation in the α-Al matrix near interfaces when the thermal residual stress surpassed its yield strength, leading to increased dislocation density at these interfaces and blocking dislocation motion. Consequently, this induced thermal mismatch strengthening and further enhanced the strengthening effect of SZMO and ZMMO NTE ceramic particles. However, when the reinforcement content exceeded 8.5 vol%, agglomerated SZMO and ZMMO NTE ceramic particles reduced their distribution uniformity within the α-Al matrix, weakening their dispersion strengthening effect while also creating a relatively high stress concentration. This increased the probability of crack initiation and propagation near aggregated NTE ceramic particles, ultimately resulting in premature failure and decreased strength and ductility for these composites.

### 3.4. Thermal Expansion Properties of SZMO-ZMMO/Al-20Si Composites

**Figure 8:**
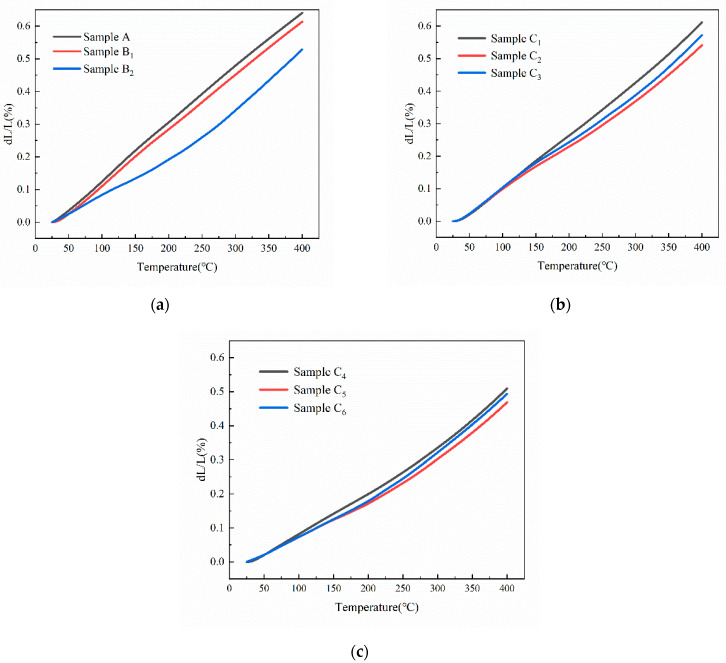
The relative length change curves of (**a**) Reference samples (Sample A, Samples B_1_ and B_2_), (**b**) Experimental samples with 5 vol% ZMMO (Samples C_1_, C_2_, C_3_) and (**c**) Experimental samples with 10 vol% ZMMO (Samples C_4_, C_5_, C_6_) in the range of room temperature to 400 °C.

**Figure 9:**
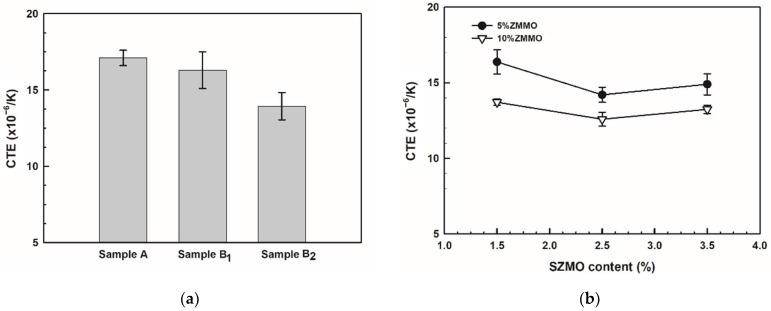
The average CTE of (**a**) Reference samples (Sample A, Samples B_1_ and B_2_), (**b**) Experimental samples (Samples C_1_ to C_6_).

In order to investigate the effects of SZMO and ZMMO NTE ceramic reinforcements on the thermal expansion properties of SZMO-ZMMO/Al-20Si composites, the relative length change curves of SZMO-ZMMO/Al-20Si composites with varying reinforcement content were measured in the temperature range of room temperature to 400 °C, and the results are shown in Figure 8. The average CTE of the SZMO-ZMMO/Al-20Si composites was estimated using the slopes from the relative length change curves, and the results are shown in Figure 9. For comparison purposes, the relative length change curve and average CTEs of reference samples (Al-20Si alloy, SZMO/Al-20Si, and ZMMO/Al-20Si composites) are also shown in Figure 8 and Figure 9. Referring to Figure 9a, it could be observed that singly adding 5 vol% SZMO NTE ceramic resulted in a mere 5.1% decrease in the CTE of Al-20Si alloy, while singly incorporating 10 vol% ZMMO NTE ceramic led to a significant decrease of up to 18.10% in the CTE. This suggested that ZMMO NTE ceramic reinforcements effectively inhibited the thermal expansion behavior of Al-20Si compared to the relatively weaker influence provided by SZMO NTE ceramic reinforcements. Referring to Figure 9b, it was evident that the combined addition of SZMO-ZMMO NTE ceramic reinforcements led to a lower CTE than the bare Al-20Si matrix, indicating an inhibition effect on CTE resulting from their incorporation. With increasing SZMO content for a certain ZMMO content, there was a decline followed by an increase in CTEs. However, increasing ZMMO content from 5 vol% to 10 vol% led to a decrease in CTE. The lowest CTE, which is 12.55 × 10^−6^/K, was achieved when combining 2.5 vol% SZMO and 10 vol% ZMMO (sample C_5_), resulting in a decrease of 26.44% compared to the bare Al-20Si alloy and a decrease of 10% compared to the ZMMO/Al-20Si composite. These findings suggested that the combined addition of SZMO and ZMMO NTE ceramic reinforcements effectively reduced the CTE in the Al-20Si alloy, thereby enhancing its dimensional stability.

The thermal expansion behavior of SZMO-ZMMO/Al-20Si composites is influenced by the thermal expansion characteristics of NTE ceramic reinforcements and the Al-20Si matrix, the interface bonding of NTE ceramic reinforcement to α-Al matrix, as well as the thermal mismatch stress at interfaces between Al-20Si matrix and NTE ceramic reinforcements. The opposing thermal expansion behaviors of the NTE ceramic reinforcement and α-Al matrix resulted in compensation during heating, where the positively CTE-induced expansion deformation of the α-Al matrix was counteracted by the negatively CTE-induced contraction deformation of the NTE ceramic reinforcements. This led to reduced CTEs in SZMO/Al-20Si, ZMMO/Al-20Si, and SZMO-ZMMO/Al-20Si composites. It can be seen from Figure 9a that since ZMMO NTE ceramic had a more negative CTE than SZMO NTE ceramic (at room temperature of 400 °C), its compensatory effect on α-Al matrix expansion deformation was stronger. Consequently, ZMMO/Al-20Si composites exhibited smaller CTEs compared to SZMO/Al-20Si composites, and the CTEs of SZMO-ZMMO/Al-20Si composites decreased with the increase in ZMMO content.

Additionally, the significant difference in CTE between the Al-20Si matrix and the ZMMO/SZMO NTE ceramic reinforcements resulted in a large thermal residual stress at the interface of SZMO/α-Al, ZMMO/α-Al, SZMO/primary Si, and ZMMO/primary Si. The magnitude of this thermal mismatch stress was influenced by the distribution and particle sizes of SZMO and ZMMO NTE ceramic reinforcements. Smaller particle sizes and a more uniform distribution in the matrix led to lower levels of thermal mismatch stress between the NTE ceramic reinforcements and the α-Al matrix. When this thermal mismatch stress was below the yield strength of the α-Al matrix during the heating, only elastic deformation occurred at their interface. The release of thermal mismatch stress depended on the elastic deformation of the α-Al matrix at the interface, resulting in a low CTE for SZMO-ZMMO/Al-20Si composites. Conversely, if the thermal mismatch stress exceeded the yield strength of the α-Al matrix, plastic deformation occurred at their interface during the heating. This release of thermal mismatch stress through plastic deformation ultimately resulted in a large CTE for SZMO-ZMMO/Al-20Si composites. Furthermore, SZMO and ZMMO NTE ceramic particles provided a dispersion-strengthening effect that increased both yield strength and plastic deformation resistance of the α-Al matrix. Consequently, the thermal mismatch stress at their interface could be released through the elastic deformation or slight plastic deformation occurring within these regions. This significantly reduced the CTE of SZMO/Al-20Si, ZMMO/Al-20Si, and SZMO-ZMMO/Al-20Si composites.

As aforementioned in the previous section, changing the volume fraction of reinforcements resulted in variations in particle size and distribution of ZMMO, SZMO, and NTE ceramic particles within an α-Al matrix. Increasing the content of NTE ceramic reinforcements led to the formation of fine and uniformly dispersed particles, which not only enhanced the dispersion-strengthening effect of NTE ceramic reinforcements on the α-Al matrix but also increased the interface areas between NTE ceramic reinforcement/α-Al. The strengthened α-Al matrix improved its resistance to deformation near the interface, while the increased interface area made the thermal mismatch stress distribution in the matrix more uniform and avoided stress concentration at the position of the reinforcement body. Both factors contributed to weakening the thermal expansion behavior of the α-Al matrix, consequently leading to a decrease in CTE for SZMO-ZMMO/Al-20Si composites. However, a high volume fraction of NTE ceramic reinforcements caused severe agglomeration of reinforcement particles within composites, diminishing their dispersion strengthening effect on the α-Al matrix, reducing the interface area between the NTE ceramic reinforcement particles and the matrix, and deteriorating the interface bonding between the NTE ceramic reinforcement particles and the matrix. This resulted in decreased resistance to deformation of the α-Al matrix and increased thermal mismatch stress at the interface. Furthermore, weak interfacial bonding between agglomerated NTE ceramic reinforcements and the α-Al matrix undermined their constraint effect on the expansion deformation of the α-Al matrix, leading to the release of thermal mismatch stress through the plastic rather than elastic deformation within these regions. As a result, there was a significant increase in CTE for SZMO-ZMMO/Al-20Si composites. Referring to Figure 3, agglomeration among NTE ceramic reinforcements began when their content exceeded 8.5 vol%, correspondingly causing an increase in CTE for SZMO-ZMMO/Al-20Si composites from this point onwards.

### 3.5. Synergy Effect of SZMO and ZMMO Reinforcements on Composites

**Figure 10:**
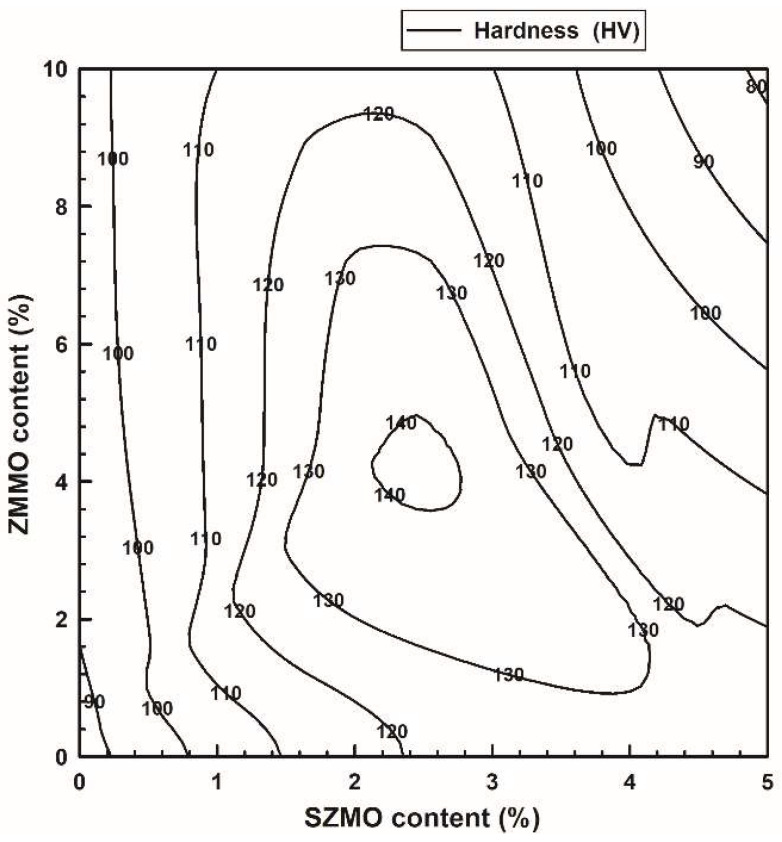
The synergy effect of SZMO and ZMMO NTE ceramic reinforcement content on the hardness of SZMO-ZMMO/Al-20Si composites.

**Figure 11:**
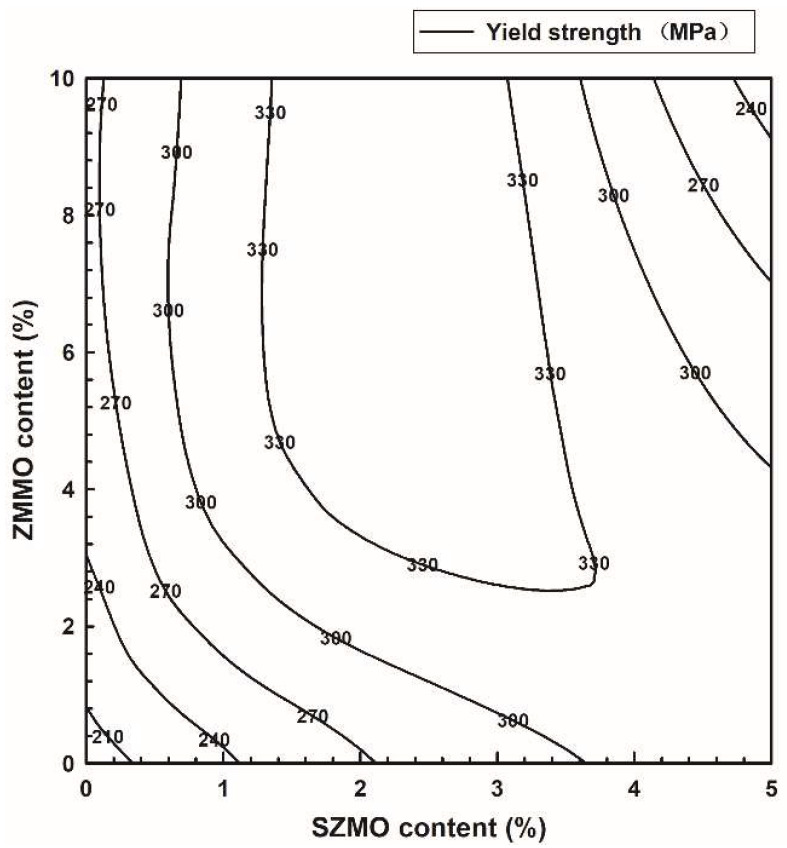
The synergy effect of SZMO and ZMMO NTE ceramic reinforcements on the compressive yield strength of SZMO-ZMMO/Al-20Si composites.

**Figure 12:**
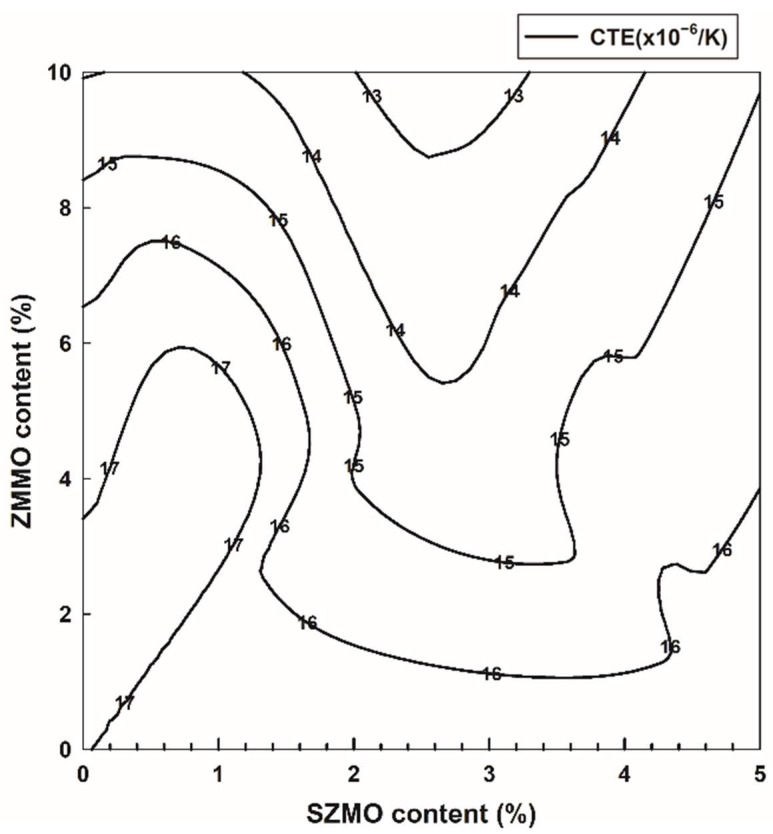
The synergy effect of SZMO and ZMMO NTE ceramic reinforcement content on CTE of SZMO-ZMMO/Al-20Si composites.

According to the previous experimental results, the combined addition of SZMO and ZMMO NTE ceramic reinforcements exhibited distinct effects on the mechanical properties and thermal expansion behavior of SZMO-ZMMO/Al-20Si composites. Adding a small amount of SZMO NTE ceramic reinforcements significantly enhanced both compressive yield strength and compressive fracture strength while exerting minimal influence on CTE in Al-20Si alloys. In contrast, the incorporation of ZMMO led to a significant decrease in CTE but had minimal influence on the compressive yield strength and fracture strengths of Al-20Si alloys. Optimal mechanical properties were achieved by adding 2.5 vol% SZMO and 5 vol% ZMMO to prepare SZMO-ZMMO/Al-20Si composites, whereas the lowest CTE was observed in ZMMO/Al-20Si composites with an addition of 2.5 vol% SZMO and 10 vol% ZMMO NTE ceramic reinforcements. In order to comprehensively evaluate the synergy effect of SZMO and ZMMO NTE ceramic reinforcements on the mechanical properties and thermal expansion behavior of SZMO-ZMMO/Al-20Si composites, a thorough analysis was conducted on hardness, compressive yield strength, and CTE. The results were then graphically presented in Figure 10, Figure 11 and Figure 12 by replotting data from Figure 5, Figure 7 and Figure 9. Referring to Figure 10 and Figure 11, it can be clearly observed that the strengthening effect of SZMO NTE ceramic exerted on the Al-20Si alloy was significantly superior to that of ZMMO NTE ceramic due to its higher strength characteristics. Only when low contents of ZMMO NTE ceramic were incorporated did the enhancing effect exerted by SZMO NTE ceramic become apparent. Figure 12 demonstrates the synergy effect between SZMO and ZMMO NTE ceramic reinforcements on the CTE in composites. It could be seen that only when high contents of ZMMO NTE ceramic were incorporated did the decrease in CTE caused by SZMO NTE ceramic become apparent due to its lower inhibitory effect on CTE characteristics.

**Figure 13:**
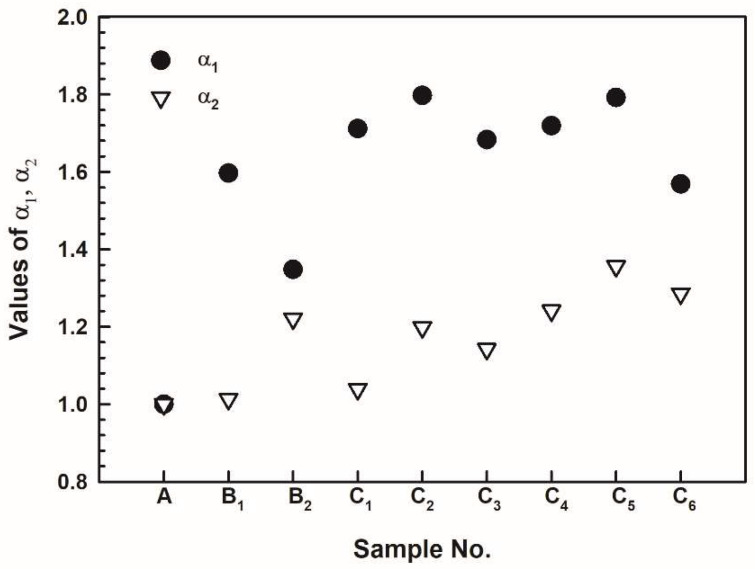
The combined evaluation of the improvement of SZMO and ZMMO NTE ceramic reinforcements on strength and CTE of SZMO-ZMMO/Al-20Si composites.

The combined analysis of Figure 10, Figure 11 and Figure 12 revealed distinct effects on the mechanical properties and CTE of SZMO-ZMMO/Al-20Si composites with a small amount of SZMO NTE ceramic reinforcements. Considering that the dimensional stability of SZMO-ZMMO/Al-20Si composites relies on not only their high yield strength but also on their low CTE, it is crucial to achieve a reasonable balance between strength and CTE in order to maintain the dimensional stability of these composites. Therefore, finding the ideal quantity for both SZMO and ZMMO NTE ceramic reinforcements necessitates considering how they affect mechanical properties as well as CTE. To address this, two parameters were proposed as expressed in Equations (3) and (4): *α*_1_ as an enhancement parameter of compressive yield strength and *α*_2_ as a reduction parameter of CTE.

(3) $$\alpha_{1}=\sigma_{ycC}/\sigma_{ycM}$$

(4) $$\alpha_{2}={CTE}_{M}/{CTE}_{\sigma }\;$$

where *σ*_ycC_ and *σ*_ycM_ are the compressive yield strengths of SZMO-ZMMO/Al-20Si composites and Al-20Si alloy, respectively, while *CTE*_C_ and *CTE*_M_ are the CTE of SZMO-ZMMO/Al-20Si composites and Al-20Si alloy, respectively. The effects of SZMO and ZMMO NTE ceramic reinforcements on the mechanical properties and thermal expansion behavior of SZMO-ZMMO/Al-20Si composites were analyzed by the parameters *α*_1_ and α_2_. Values greater than 1 for either *α*_1_ or *α*_2_ indicated desired combinations that result in improved mechanical properties or reduced CTE. Larger values of *α*_1_ corresponded to better mechanical properties of composites, while larger values of *α*_2_ corresponded to a lower CTE of composites. Figure 13 shows how the combined additions of SZMO and ZMMO NTE ceramic reinforcements affected *α*_1_ and *α*_2_. It can be clearly seen that the addition of SZMO NTE ceramic alone exhibited a superior effect on enhancing the composite’s strength, whereas ZMMO NTE ceramic alone demonstrated a greater impact on decreasing CTE. The combined addition obtained a more pronounced improvement in strength as opposed to reducing the CTE of SZMO-ZMMO/Al-20Si composites.

To determine the optimal addition amount of SZMO and ZMMO reinforcements of SZMO-ZMMO/Al-20Si composites with high dimensional stability, parameter α was proposed as expressed in Equation (5):

(5) $${\alpha =\alpha }_{1}\times \alpha_{2}$$

**Figure 14:**
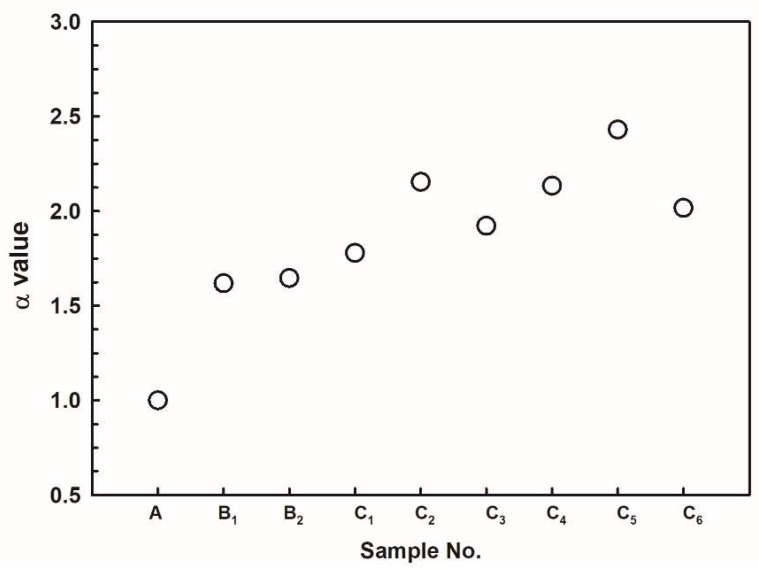
The synergy effects of SZMO and ZMMO NTE ceramic reinforcements on yield strength and CTE of composites.

The large value of α represents the desired combination of SZMO and ZMMO NTE ceramic reinforcement, which provides the optimal balance between strength and CTE for the prepared SZMO-ZMMO/Al-20Si composites. Figure 14 represents the value of *α* for all prepared SZMO-ZMMO/Al-20Si composites with varying contents of SZMO and ZMMO NTE ceramic reinforcements. It can be seen that sample C_5_, which was prepared by adding 10 vol% ZMMO in combination with 2.5 vol% SZMO, exhibited the largest α, demonstrating it possesses the most superior balance between CTE and strength. This composite had a compressive yield strength of 349.72 MPa and a CTE of 12.55 × 10^−6^/K, representing an increase in yield strength of 79.20% compared to the Al-20Si alloy while reducing CTE by 26.44%.

## 4. Conclusions

(1) SZMO-ZMMO/Al-20Si composites were fabricated by adding varying contents of SZMO and ZMMO NTE ceramic reinforcements. Both the SZMO and ZMMO NTE ceramic reinforcements exhibited excellent phase stability. However, excessive addition of those reinforcements could cause their aggregation within the composite.
(2) Adding a small amount of SZMO significantly enhanced the reinforcing effect provided by ZMMO reinforcements on the Al-20Si matrix alloy. The combined incorporation of 2.5 vol% SZMO and 5 vol% ZMMO NTE ceramic reinforcements results in an increase of 63.5% in hardness and 79.7% in compressive yield strength compared to the bare Al-20Si alloy, respectively.
(3) The addition of a small amount of SZMO in combination with ZMMO reinforcements effectively reduced the CTE in SZMO-ZMMO/Al-20Si composites. The lowest CTE achieved was 12.55 × 10^−6^/K when combining 2.5 vol% SZMO NTE ceramic and 10 vol% ZMMO NTE ceramic, resulting in a decrease of 26.44% compared to the bare Al-20Si alloy and a decrease of 10% compared to the ZMMO/Al-20Si composite.
(4) Based on the evaluation parameter proposed in this study, the composite prepared by adding 2.5 vol% SZMO NTE ceramic and 10 vol% ZMMO NTE ceramic exhibited an optimal balance between CTE and strength. This represented a significant increase in yield strength by 79.20% and a notable reduction in CTE by 26.44%, compared to the bare Al-20Si alloy.

## Figures and Tables

**Table 1:** The composition of SZMO-ZMMO/Al-20Si composites (vol%).

	Reference Samples			Experimental Samples					
	A	B_1_	B_2_	C_1_	C_2_	C_3_	C_4_	C_5_	C_6_
Al-20Si	100	95	90	93.5	92.5	91.5	88.5	87.5	86.5
SZMO	0	5	0	1.5	2.5	3.5	1.5	2.5	3.5
ZMMO	0	0	10	5	5	5	10	10	10

## Data Availability

Data are contained within the article.
